# Image of the Month: High Pressure Distal Colostogram in a Patient with an Anorectal Malformation

**DOI:** 10.1055/s-0040-1721051

**Published:** 2020-11-23

**Authors:** Anisha Apte, Giulia Brisighelli, Elise McKenna, Marc A. Levitt

**Affiliations:** 1 Department of Surgery, The George Washington University School of Medicine and Health Sciences, Washington, District of Columbia, United States; 2 Department of Surgery, Paediatric Surgery, Chris Hani Baragwanath Hospital, Johannesburg, Gauteng, South Africa; 3 Department of General and Thoracic Surgery, Children's National Medical Center, Washington, District of Columbia, United States; 4 Division of Colorectal and Pelvic Reconstruction Surgery, Children's National Medical Center, Washington, District of Columbia, United States

**Keywords:** high pressure distal colostogram, anorectal malformation, recto-urinary fistula

## Abstract

An adequately performed high pressure distal colostogram is crucial to plan surgery in male patients born with anorectal malformations. We present two male patients that underwent a divided sigmoid colostomy with distal mucus fistula in the neonatal period and at 6 months of age underwent a high pressure distal colostogram. In the discussion, we will give some tricks beyond the known rules: how to correctly interpret a high pressure distal colostogram, how to identify the level of a recto-urinary fistula, and how to accurately plan the surgical approach.

## Case Report

**Fig. 1:**
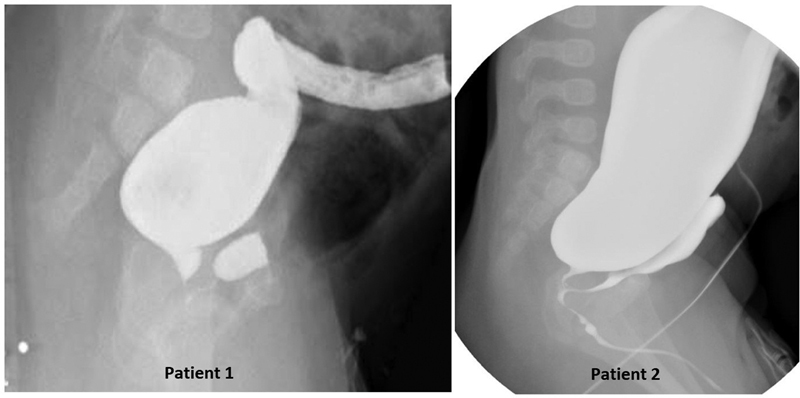
Two high pressure distal colostograms.

Two 6-month-old male infants with anorectal malformation (ARM) who underwent divided descending colostomies with distal mucus fistulae as newborns, now present to your clinic for preoperative planning for definitive repair. You review the newborns' colostograms ( Fig. 1 ) to attempt to identify the anatomy of the malformation and plan your surgical approach for repair.

## Discussion

Recto-urethral fistulas are the most common anatomic finding in male newborns with ARM. 1 The high pressure distal colostogram (or augmented pressure distal colostogram) is the technique of choice, and is standardized in most radiology departments, to help to determine the type of malformation in a male child born with imperforate anus. 2 3 Important features to assess when reviewing a high pressure distal colostogram are length of the rectum, adequate distension of the rectum, its relationships with the surrounding bony structures (especially, the sacrum and the marker located at the anal dimple), and the presence and level of a fistula with the urinary tract. 2

**Fig. 2:**
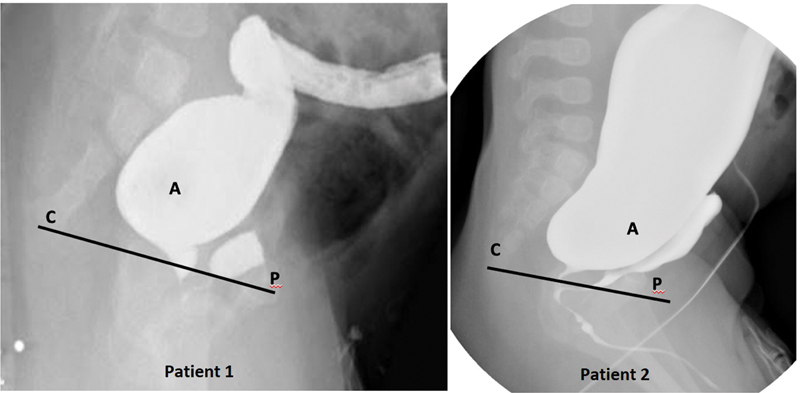
( **A** ) Rectal pouch shown above the pubococcygeal line, P = center of the pubic ossific center. C = (coccyx point) most distal ossified visible bony segment of the coccyx to help guiding the surgeon in deciding the approach. PC, pubococcygeal line.

In the two cases presented ( Fig. 1 ), the high pressure distal colostogram can be considered of adequate quality because the bony parts (sacrum, pubic symphysis, and femur heads), and a radiopaque marker in the anal dimple are visible as well as a Foley balloon to seal the mucus fistula and avoid spillage of contrast. Moreover, the entire distal bowel is filled with contrast and the rectum appears convex shaped and adequately distended. The sacrum and coccyx appear well developed, and in both images, the opacified rectum ends higher than the last bony segment (above the pubococcygeal [PC] line; Fig. 2 ). 4 A fistula with the urinary tract can be visualized, and the bladder and part of the urethra also appear to be partially filled with contrast.

**Fig. 3:**
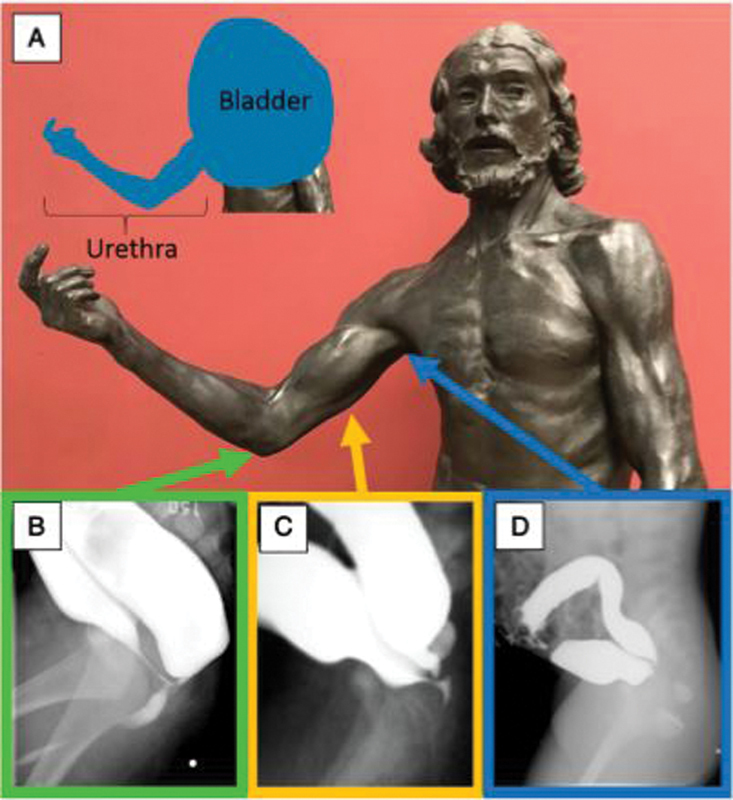
Auguste Rodin's Saint John the Baptist sculpture (1880) in the Musée d'Orsay, Paris, France. The right arm is used to represent the course of the male urethra ( **A** ). The relative positions of the male urethra are represented by the various levels of the arm of the statue. The elbow represents the bulbar urethra ( **B** ), the humerus represents the prostatic urethra ( **C** ), and the axilla represents the bladder neck ( **D** ). Reprinted with permission from Halleran et al. 3

Recto-urinary fistulas can be further classified as recto-bulbar urethral fistulas, recto-prostatic urethral fistulas, and recto-bladder neck fistulas. Recto-bladder neck and recto-prostatic fistulas tend to lie high above the coccyx, while recto-bulbar fistulas tend to lie just beneath the levator ani. 1 This is significant because while recto-bulbar fistulas are easily accessed via an extraperitoneal posterior approach, the coccyx and sacrum may limit exposure and creates for a challenging dissection for those fistulas located above it. In these circumstances, a transabdominal approach (laparoscopy or laparotomy) might be necessary to expose and gain access to the fistula. 5 A very good trick on how to determine the level of the recto-urinary fistula has been suggested by Halleran et al ( Fig. 3 ). 3 The right arm can be used to represent the course of the male urethra: with the elbow representing the bulbar urethra, the humerus representing the prostatic urethra, and the axilla representing the bladder neck. The recto-urethral fistula can insert at any of those levels, and therefore, it can be a recto-bulbar, recto-prostatic, or recto-bladder neck fistula. The high pressure distal colostograms presented in these cases show a recto-urethral-prostatic fistula ( Fig. 1 , patient 1) and a recto-urethral bulbar fistula ( Fig. 1 , patient 2).

Because it is not always easy to obtain good quality images with all elements contrasted, another trick can be used to guide the pediatric surgeon in determining the level of the recto-urinary fistula: the ease with which the bladder fills with contrast. During the high pressure distal colostogram, if the contrast easily and preferentially fills the bladder, the fistula must be located above the urinary sphincter and is most likely a recto-urethral prostatic, or recto-bladder neck fistula. If the contrast goes preferential to the distal urethra and filling the bladder is difficult, the fistula is most likely distal to the urinary sphincter and hence, at the bulbar urethral level.

Approximately 90% of male ARM defects can be repaired with a PSARP, and transabdominal approach (via lapaorotomy or laparoscopy) should only be performed for recto-bladder neck fistulas and high recto-prostatic fistulae that are located above the coccyx. 1 This is because, for a recto-bladder neck fistula, dissecting the rectum off the bladder can usually be easily accomplished, given that the two structures do not share much of a common wall. Recto-bulbar fistulas on the other hand, tend to have a longer and thinner wall between the rectum and urethra, making laparoscopic dissection very technically challenging and increasing the risk of damaging nearby structures, including the urethra, bladder neck, vas deferens, and seminal vesicles. Additionally, an incomplete dissection of the distal fistula or rectum could result in recurrent fistula or a leaving behind a remnant of the original fistula (R.O.O.F.), respectively. 5

The ultimate aim of the high pressure distal colostogram is to help the surgeon determine if the ARM should be approached via a transabdominal approach or via perineal approach only. 2 A trick for this based on the contrast study is by drawing the PC line and determining what is the first structure you will find when the surgical approach is via a posterior sagittal incision ( Fig. 3 ). 3 4 If the most posterior structure is the rectum, then a posterior sagittal approach only is deemed safe, and if it is the urinary tract, then laparoscopy or laparotomy is preferred. To make this decision, it is also helpful to look at the bulkiness of the distal bowel. If the rectum is very bulky, a laparoscopic approach to reach the fistula might be difficult to perform and therefore a posterior sagittal approach only might be indicated. 3 The surgical decision, however, depends also on the expertise of the surgeon, and therefore, it is necessary to keep in mind that different surgeons might prefer different approaches and it is not always easy to find agreement and to create universal protocols.

The following goals must be achieved:

- The distal rectum must be dissected fully, so as not to leave behind a remnant of the original fistula (R.O.O.F.), but not too aggressively so as to injure the urinary tract.
- The fistula might be ligated.
- The rectum mobilized to the anoplasty with good blood supply and without tension.

The ultimate goal is to use the high pressure distal colostogram to obtain as much information as possible and to then plan the surgery based on the information and on the and experience of the surgeon, aiming to perform the safest procedure for the child. When asking a panel of colorectal experts what approach they would have used to treat patient 1 in Fig. 1 , 63% of them suggested a PSARP only approach and 37% of them would have used laparoscopy to separate the fistula, confirming that there is no right answer. For the case in patient 2 in Fig. 1 , the risk of injuring the urethra and other critical structures during posterior dissection with the PSARP made initial transabdominal dissection and mobilization of the rectum the preferred choice for most experts. To quote one of our professors: “The operation you should do is the one you do best.”

## Conclusion

Besides the known rules to perform and interpret a high pressure distal colostogram, additional tricks can be used to obtain more information. Comparing the urethra to the parts of the arm can help identify the level of the fistula ( Fig. 3 ). Assessing how easily the contrast fills the bladder and the proximal urethra also provides a useful trick if the fistula is above or below the urinary sphincter. Finally, drawing a PC line can help the surgeon decide what surgical approach is preferable. Patients with rectourethral fistulas located above the coccyx or rectal pouch located above the PC line should be evaluated for a laparoscopic approach because of the potential for injury to the urethra and other surrounding structures during an extensive posterior dissection. The ultimate goal is to plan the surgical repair according to the surgical experience, putting the safety of the child first.

## Notes

New Insights and the Importance for the Pediatric Surgeon Tricks to determine the location of a recto-urethral fistula:

- Using the right arm to represent the course of the male urethra. If the fistula is visualized at the elbow or lower, it would be recto-bulbar; at the humerus level, it would be recto-prostatic; and at the axilla or above, it is in keeping with a recto-bladder neck fistula.
- The easiness to fill the bladder with contrast can give you a hint about the level of the fistula: if you can fill preferentially the bladder the fistula is above the urinary sphincter and most likely a bladder neck fistula. If the contrast goes preferential to the distal urethra and filling the bladder is difficult, it is most likely distal to the urinary sphincter hence bulbar.

Tricks to decide on surgical approach:

- Drawing a pubococcygeal (PC) line to determine what is the first structure you will find when you open posterior sagittally: if it is rectum, then it is deemed safe, and if it is urinary tract, then laparoscopy or laparotomy should be preferred.
- These tools can help the pediatric surgeon correctly interpret a high pressure distal colostogram and the level of a rectourinary fistula to plan the right surgical approach for reconstruction.
